# Seronegative Rheumatoid Arthritis Secondary to Immune Checkpoint Inhibitor in a Renal Cell Carcinoma Patient Encountered in a Rural Community-Based Rheumatology Clinic: A Case Report

**DOI:** 10.7759/cureus.41394

**Published:** 2023-07-05

**Authors:** Elmer R De Camps Martinez, Camila Gonzalez, Hamzah Hassan, Hafsa Hassan, Farooq Hassan

**Affiliations:** 1 Internal Medicine, Northeast Ohio Medical University, Rootstown, USA; 2 Genomic Medicine Institute, Cleveland Clinic, Cleveland, USA; 3 Rheumatology, Trumbull Regional Medical Center, Warren, USA

**Keywords:** renal cell carcinoma, seronegative rheumatoid arthritis, immune-related adverse event (irae), nivolumab, immune checkpoint inhibitors (icis)

## Abstract

Immune checkpoint inhibitors (ICIs) are a widely used class of cancer immunotherapy. Those drugs have improved the treatment of cancer since its introduction in the 2000s. Nivolumab is an ICI that can be used for previously untreated renal cell carcinoma. Immune-related adverse events (irAEs) are a type of adverse event of immunotherapy that is associated with an overreactive immune system. We report a case of a 69-year-old Caucasian man with stage IV renal cell carcinoma who presented to a rural community-based rheumatology clinic referred by his oncologist, after starting to develop morning stiffness for at least three hours, joint swelling, warmth, and erythema five months after starting immunotherapy with nivolumab. The patient was diagnosed with seronegative rheumatoid arthritis secondary to ICIs and required a higher dose of prednisone (up to 40 mg per day) with methotrexate to achieve remission. With the widespread availability of ICIs, rheumatologic irAEs can be encountered in a rural community-based practice. Practicing physicians taking care of cancer patients need to be aware of the adverse effect of ICIs.

## Introduction

Immune checkpoint inhibitors (ICIs) are a widely used class of cancer immunotherapy. In the modern landscape of medicine, there are a wide variety of ICIs available for the treatment of various malignancies including metastatic melanoma, renal cell carcinoma (RCC), and non-small cell lung cancer, among others. ICIs as a drug class work by modifying the host response to enhance the physiological antitumor response mediated by T cells, thus eliminating malignant cells at a greater magnitude. Nivolumab has improved the treatment of cancer since it was first approved for unresectable or advanced melanoma by the United States Food and Drug Administration (FDA) in 2014. Nivolumab, which is a programmed cell death protein 1 (PD-1) receptor antagonist that is part of ICIs was FDA approved for previously untreated advanced RCC in 2018 [1].

Nivolumab results in inhibition of the cancer immune escape pathway in turn leading to a greater physiologic immune response against cancer cells. ICIs can lead to off-target tissue damage known as immune-related adverse events (irAEs) due to nonspecific T-cell activation. Rheumatological irAEs have been scarcely reported in the published literature when compared to other types of irAEs. We report a case of seronegative rheumatoid arthritis (RA) encountered in a rural community-based rheumatology clinic after starting nivolumab, that was successfully treated with discontinuation of ICIs, prednisone, and methotrexate.

## Case presentation

A 69-year-old Caucasian man with a history of non-insulin-dependent type 2 diabetes mellitus and stage IV RCC presented to a rural community-based rheumatology clinic referred by his oncologist for evaluation of chronic symmetric inflammatory arthritis involving both hands.

A year and five months before the current presentation, the patient was diagnosed with stage IV RCC involving both lungs and initially started on frontline immunotherapy with ipilimumab and nivolumab. After receiving four cycles of ipilimumab and nivolumab, it was subsequently changed to nivolumab/cabozantinib due to ICIs induced colitis which responded to prednisone 20 milligram (mg) twice per day and discontinuation of ipilimumab.

**Figure 1:**
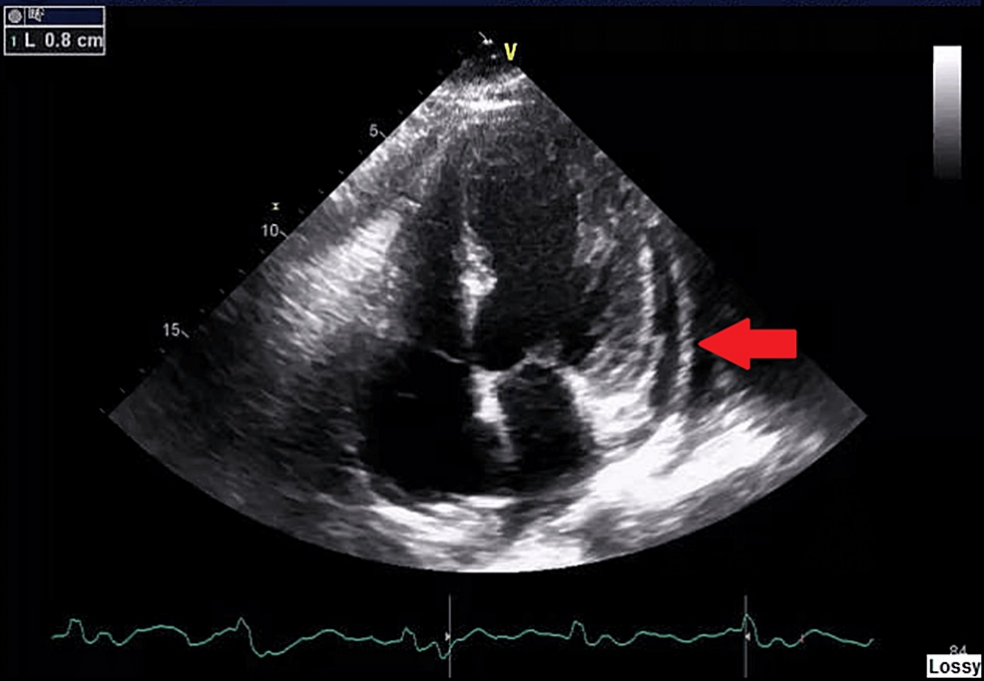
A4C view of 2D TTE showing a small pericardial effusion. A4C: Apical four-chamber, 2D: Two dimension, TTE: Transthoracic echocardiogram

A year before the current presentation, the patient developed an itchy erythematous rash over his extremities that resolved with a second course of prednisone with its subsequent taper. Ten months before the current presentation, the patient started to develop bilateral knee arthralgia for which he got intra-articular glucocorticoid injection by his orthopedic surgeon, which did help with the arthralgia and was started again on prednisone 5 mg daily. Eight months before the current presentation, the patient had worsening polyarthralgia and myalgias now in all his extremities, his prednisone was increased from 5 mg to 10 mg daily and the cabozantinib was held but nivolumab was continued. Seven months before the current presentation the cabozantinib was held, and prednisone 10 mg per day was continued. The patient got a transthoracic echocardiogram from his cardiologist that showed a left ventricular ejection fraction of 35% with moderate pericardial effusion (Figure 1) and was diagnosed with heart failure with reduced ejection fraction (HFrEF) for which he was started on guideline-directed medical therapy (GDMT).

Five months before the current presentation, the patient had significant improvement with the prednisone and holding the cabozantinib. The patient got a left heart catheterization from his cardiologist that did not show significant stenosis of the coronary arteries.

Three months before the current presentation, the patient had COVID-19 and was treated with nirmatrelvir/ritonavir as outpatient.

Two months before the current presentation, the patient restarted cabozantinib concurrently with prednisone 10 mg, but after five days with diarrhea, cabozantinib was stopped. The patient was started on medical marijuana for decreased appetite and weight loss.

One month before the current presentation, the patient was still complaining of polyarthralgia and myalgias, and his prednisone was increased to 20 mg daily.

Two weeks before the current presentation, the patient could not taper off the prednisone 20 mg daily due to persistent polyarthralgia and myalgias thus the patient was referred to the rheumatology clinic for additional evaluation.

In the rheumatology clinic, the patient presented with synovitis involving his hands, wrists, elbows, and shoulders. He has been having difficulty getting up from his chair and getting up from the toilet. He also has been having morning stiffness lasting about three to four hours. He had been having weight loss and loss of appetite. He stated that his prednisone was recently increased from 10 mg daily to 20 mg daily, but he mentioned that he has not noticed any improvement in his symptoms with the prednisone. He denies Raynaud’s phenomenon, photosensitivity, alopecia, and recurrent oral ulcers. He has been having difficulty doing his daily living activities.

**Figure 2:**
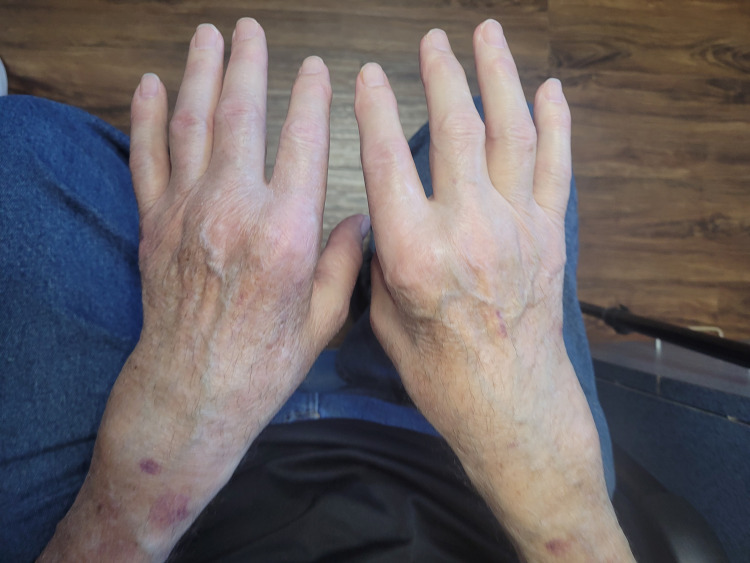
Synovitis of both hands at presentation.

On examination, the heart rate was 84 beats per minute, the blood pressure was 134/70 mm Hg, and the oxygen saturation was 97% while the patient was breathing ambient air. He weighed 99 kg and the body-mass index was 32.19. He was alert and oriented, the lung fields were clear, and there were no heart murmurs. There was an erythematous malar rash with overlying red papules sparring the nasolabial fold, severely active synovitis was noticed on metacarpophalangeal (MCP) joints, proximal interphalangeal (PIP) joints and radiocarpal joint bilaterally (Figure 2). No significant synovitis was noted in the patient’s ankles and feet. A good range of motion was noticed in all patient joints. Muscle power was four out of five in both proximal and distal muscles. Patrick’s maneuver was negative and Schober’s test was within normal limits. Diffuse tender points were noted on the bilateral lateral epicondylar region, mid trapezius, trochanter area, and the medial fat pad of the bilateral knees. The remainder of the examination was normal. Initial laboratory results are shown in Table 1.

**Table 1:** Initial laboratory tests

Test	Value	Reference range and units
White Blood Cell	9.9	3.8-10.8 thousands/µL
Hemoglobin	11	13.2-17.1 g/dL
Hematocrit	34.7	38.5-50%
Mean Corpuscular Volume (MCV)	80.7	80-100 fL
Red Blood Cell Distribution Width (RDW)	15.2	11%-15%
Platelet (PLT) count	357	140-400 thousands/µL
Absolute Neutrophils Count	8682	1,500-7,800 cells/µL
Absolute Lymphocyte Count	891	850-3,900 cells/µL
Absolute Monocytes Count	20	15-500 cells/µL
Absolute Eosinophils Count	20	15-500 cells/µL
Absolute Basophils Count	30	0-200 cells/µL
Erythrosedimentation rate (ESR)	77	<20 mm/h
C-reactive protein	65	<8.0 mg/L
Total protein	6.8	6.1-8.1 g/dL
Albumin	3.4	3.8-4.8 g/dL
Alpha 1 Globulin	0.5	0.2-0.3 g/dL
Alpha 2 Globulin	1	0.5-0.9 g/dL
Beta 1 Globulin	0.4	0.4-0.6 g/dL
Beta 2 Globulin	0.5	0.2-0.5 g/dL
Gamma Globulin	1	0.8-1.7 g/dL
Aspartate aminotransferase (AST)	7	10-35 g/dL
Alanine aminotransferase (ALT)	7	9-46 g/dL
Creatinine	0.78	0.70-1.30 mg/dL
Estimated Glomerular Filtration Rate (eGFR)	103	>60 mL/min/1.73m^2^
Uric Acid	6.2	4-8 mg/dL
Cold Hemagglutinins	None Detected	None Detected Titer
Thyroid Stimulating Hormone (TSH)	1.02	0.40-4.50 mIU/L
Calcium	9.3	8.6-10.3 mg/dL
Complement Component C3C	176	82-185 mg/dL
Complement Component C4C	37	15-53 mg/dL
Aldolase	5.2	<8.1 U/L
Creatinine Kinase (CK)	39	44-196 U/L
Vitamin B12	305	193-986 mg/dL
Aldose	5.2	Less or equal 8.1 U/L
Urine Microalbumin	0.85	
Urine Creatinine	85	
Microalbumin/Creatinine ratio	<10	0-30 mcg/mg
Hemoglobin A1c	6.2	4%-5.6%
25-Hydroxy Vitamin D	51.6	32-100 ng/mL

An electromyography and nerve conduction study of each upper extremity and each lower extremity showed bilateral focal median neuropathy at the wrist and sensorimotor polyneuropathy with both axonal and demyelinating features on the upper extremities and sensorimotor polyneuropathy, axonal type on lower extremities. The study was negative for spontaneous activity or myopathic potentials.

X-rays of his hands, wrist, and pelvis just showed degenerative disease, there was no evidence of erosions. Extensive immunologic laboratory workup was done which was mostly negative except for the anti-melanoma differentiation-associated protein 5 (anti-MDA5) antibody, which was positive (Table 2).

**Table 2:** Immunologic laboratory tests Ig: Immunoglobulin, MDA5: Melano Differentiation-Associated Protein 5; TNF: Tumor Necrosis Factor, NXP: Nuclear Matrix Protein

Test	Value	Reference range and units
HLA-B27 Antigen	Negative	Negative
Anti-cytrullinate antibody (Anti-CCP)	5	0-19 units
Antinuclear antibody (ANA)	Negative	Negative <1:80 titers
Single stranded DNA (ssDNA)	53	0-99 Units/mL
Double stranded DNA (dsDNA)	2	0-40 Units/mL
Anti-Smith	0	0-89 Units/mL
Anti-Ro	0	0-91 Units/mL
Anti-La	4	0-73 Units/mL
Anti-Chromatin	5	0-99 Units/mL
Anti-Scl-70	0	0-32 Units/mL
Anti-centromere	3	0-100 Units/mL
Rheumatoid Factor IgM	0	0-25 Units/mL
Rheumatoid factor IgG	9	0-20 Units/mL
Rheumatoid factor IgA	0	0-35 Units/mL
Anti-CCP IgG	0.2	0-5 Units/mL
Anti-Thyroid Peroxidase (Anti-TPO)	1	0-5 Units/mL
Anti-cardiolipin antibody (aCL) IgM	3	0-10 MPL Units/mL
aCL IgG	1	0-15 GPL Units/mL
aCL Ig A	0	0-7 APL Units/mL
Anti-beta 2 glycoprotein Antibody IgM	0	0-4 Units/mL
Anti-beta 2 glycoprotein antibody IgG	1	0-25 Units/mL
Anti-beta 2 glycoprotein antibody IgA	0	0-4 Units/mL
Interferon Gamma immunoassay	Negative	Negative
Anti-Jo-1 antibody	<11	<11 SI
Anti-PL-7 antibody	<11	<11 SI
Anti-PL-12 antibody	<11	<11 SI
Anti-EJ Antibody	<11	<11 SI
Anti-EJ Antibody	<11	<11 SI
Anti-OJ Antibody	<11	<11 SI
Anti-SRP antibody	<11	<11 SI
Anti-MI-2 Alpha Antibody	<11	<11 SI
Anti-MI-2 Beta Antibody	<11	<11 SI
Anti-MDA-5 Antibody	16	<11 SI
Anti-TNF1-gamma	<11	<11 SI
Anti-NXP-2 Antibody	<11	<11 SI

The patient was advised to increase the prednisone to 40 mg daily for two weeks and then reduce it by 5mg every two weeks until it reaches 20 mg daily. One month after, he continued to have extensive synovitis with mild improvement and was kept on nivolumab with tivozanib. A diagnosis of seronegative RA was made, and the patient was advised to discuss the use of methotrexate with his oncologist as well as continue the prednisone. A month after, the patient was admitted to a rehabilitation center while trying to taper his prednisone due to a RA flare and was discharged on methotrexate 12.5 mg per week with folic acid.

The patient followed up one month after discharge, nivolumab was discontinued but tivozanib was continued by his oncologist. He had significant improvement in his synovitis as well as his inflammatory markers with methotrexate and prednisone (Table 3). Methotrexate was increased to 17.5 mg per week with a gradual increase to a target of 22.5 mg per week.

**Table 3:** Inflammatory markers trend ESR: Erythrosedimentation rate, CRP: C-reactive protein

Test	On presentation	After one month	After two months	Reference range and units
ESR	77	34	20	0-20 mm/hr
CRP	6.5	0.40	0.32	0.00-0.30 mg/dL

## Discussion

ICIs are a type of cancer immunotherapy that works by inhibiting immune checkpoints with biological agents. Normally, the immune checkpoints cytotoxic T-lymphocyte associated protein-4 (CTLA-4), and PD-1 pathways, downregulate T-cell responses and act to protect the body from the overactive immune response. By inhibiting those pathways, the T-cell destroys the cancer cells [2,3]. There are several biologic agents approved by the FDA for numerous types of cancer, such as Ipilimumab (anti-CTLA-4 antibody) and nivolumab (anti-PD-1 antibody), which were used in our patient.

IrAEs are a type of adverse event of immunotherapy that is associated with an overreactive immune system. Rheumatologic and musculoskeletal irAE incidence has not been clearly established but in a meta-analysis, it was stated that the prevalence was 6.13% [4,5].

The pathogenesis for the development of irAEs is an area of research, to this date there is no available correlative date for the rheumatologic irAEs. A proposed mechanism is that ICIs result in non-specific upregulation of immune pathways. PD-1 inhibition increases T-cell activation and proliferation, abrogates regulatory T-cells (Tregs) functions, and boosts humoral autoimmunity, although it is likely that specific pathways are prominent in one therapy type versus the others, resulting in differences in irAE phenotypes. PD-1 inhibition enhances T-cell activation but results in different phenotypes of irAE than CTLA-4 inhibition. PD-1 is expressed on T cells, whereas its ligands PD-L1 and PD-L2 are present on antigen-presenting cells, tumor cells and various normal tissues, and normally act to downregulate T-cell activation. Both PD-1 and PD-L1 are expressed by Tregs, and this pathway seems to be involved in the differentiation of T helper type 1 (Th1) cells into Tregs. Mice deficient in PD-1 or PD-L1 develop various autoimmune manifestations depending on their genetic backgrounds, which, in some cases is mediated by autoantibodies [3].

RA is a common immune-mediated disease that is primarily characterized by symmetric, polyarticular inflammatory arthritis, typically involving small joints of hands and feet. The diagnosis of RA is made using the 2010 American College of Rheumatology (ACR)/European Alliance of Associations for Rheumatology (EULAR) classification criteria for RA which requires six or more points to classify a patient with RA. Our patient met seven points given his small joint involvement of more than 10 (five points), abnormal CRP or ESR (one point), and duration of more than six weeks (one point) in the setting of negative anti-CCP and rheumatoid factor which confer a diagnosis of seronegative RA [6].

According to the American Society of Clinical Oncology (ASCO) for the management of irAEs in patients treated with ICI therapy, this patient was cataloged as a grade three to grade four musculoskeletal toxicity of ICI therapy. Because the patient had severe synovitis that was interfering with his self-care activities of daily living and could not taper off prednisone to less than 10 mg per day and required methotrexate [7]. The patient responded to the therapy as evidenced by his decreased synovitis and down trending of the inflammatory markers.

There have been two case reports describing seronegative RA induced by ICI, one of them in Osaka, Japan, and the other one in Kansas, United States of America (USA). Both were in an urban university-based hospital [8,9]. We could not find any case described in a rural community-based hospital. This case highlighted the necessity of vigilantly monitoring for irAE in receiving immunotherapy with ICI, clinician should be aware of the possibility of encountering such cases in rural areas where immunotherapy has reached.

## Conclusions

ICIs have revolutionized cancer treatment, but they also pose a possibility of irAEs, such as RA. The case highlights the necessity of vigilant monitoring for irAEs in receiving immunotherapy and the possibility of encountering such cases in rural community practices. Seronegative RA secondary to ICI is a rare irAEs that should be kept in mind in patients receiving ICI therapy. Further research is required to determine the incidence, risk factors, pathogenesis, and optimal approach for managing seronegative RA secondary to ICI therapy.
